# *M*^3^: using mask-attention and multi-scale for multi-modal brain MRI classification

**DOI:** 10.3389/fninf.2024.1403732

**Published:** 2024-07-29

**Authors:** Guanqing Kong, Chuanfu Wu, Zongqiu Zhang, Chuansheng Yin, Dawei Qin

**Affiliations:** ^1^Linyi People's Hospital, Linyi City, Shandong Province, China; ^2^Linyi Key Laboratory of Health Data Science, Linyi City, Shandong Province, China; ^3^Shandong Open Laboratory of Data Innovation Application, Linyi City, Shandong Province, China

**Keywords:** brain tumor classification, HT prediction in stroke, deep learning, attention mechanism, multi-scale feature fusion

## Abstract

**Introduction:**

Brain diseases, particularly the classification of gliomas and brain metastases and the prediction of HT in strokes, pose significant challenges in healthcare. Existing methods, relying predominantly on clinical data or imaging-based techniques such as radiomics, often fall short in achieving satisfactory classification accuracy. These methods fail to adequately capture the nuanced features crucial for accurate diagnosis, often hindered by noise and the inability to integrate information across various scales.

**Methods:**

We propose a novel approach that mask attention mechanisms with multi-scale feature fusion for Multimodal brain disease classification tasks, termed *M*^3^, which aims to extract features highly relevant to the disease. The extracted features are then dimensionally reduced using Principal Component Analysis (PCA), followed by classification with a Support Vector Machine (SVM) to obtain the predictive results.

**Results:**

Our methodology underwent rigorous testing on multi-parametric MRI datasets for both brain tumors and strokes. The results demonstrate a significant improvement in addressing critical clinical challenges, including the classification of gliomas, brain metastases, and the prediction of hemorrhagic stroke transformations. Ablation studies further validate the effectiveness of our attention mechanism and feature fusion modules.

**Discussion:**

These findings underscore the potential of our approach to meet and exceed current clinical diagnostic demands, offering promising prospects for enhancing healthcare outcomes in the diagnosis and treatment of brain diseases.

## 1 Introduction

The brain, the most sophisticated organ in the nervous system with over a hundred billion neurons, plays a pivotal role in controlling bodily functions. Aberrations in brain function, particularly brain tumors and Hemorrhagic Transformation (HT) post-stroke treatment, are critically detrimental to human health (Louis et al., 2016; Virani et al., 2021).

**Figure 1:**
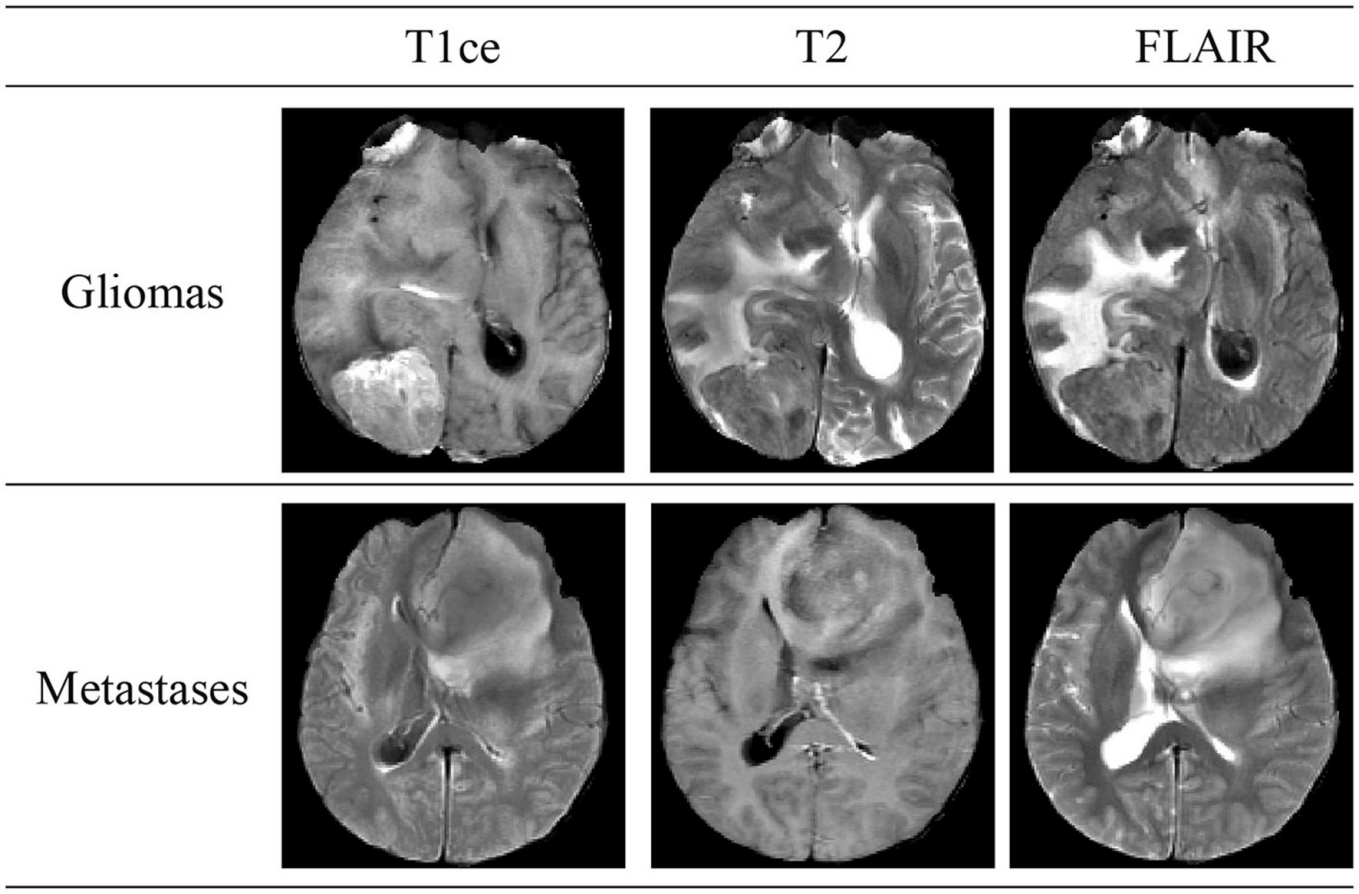
Schematic diagrams illustrating the brain structures of patients with glioma and brain metastasis across three MRI modals. The details of three modals can be found in Section 2.1.

Brain tumors, classified into primary and secondary (metastatic) types, are a significant health threat due to their potential to damage the nervous system and endanger lives through tissue compression. Gliomas, constituting 40–50% of primary intracranial tumors, are the predominant type of primary brain tumors. Secondary, or metastatic, brain tumors occur when cancer cells spread from other body parts to the brain, manifesting symptoms akin to primary brain tumors. Figure 1 illustrates schematic diagrams of gliomas and metastatic tumors under the three modalities of T1ce, T2, and FLAIR. Differentiating between metastatic brain tumors with unknown primary origins and gliomas presents a clinical challenge, particularly when patients exhibit similar symptoms, signs, and radiological features. Such diagnostic ambiguity may delay treatment and increase the risk of further metastasis or recurrence, underscoring the necessity for advancements in differential diagnosis through imaging techniques (Tandel et al., 2019).

**Figure 2:**
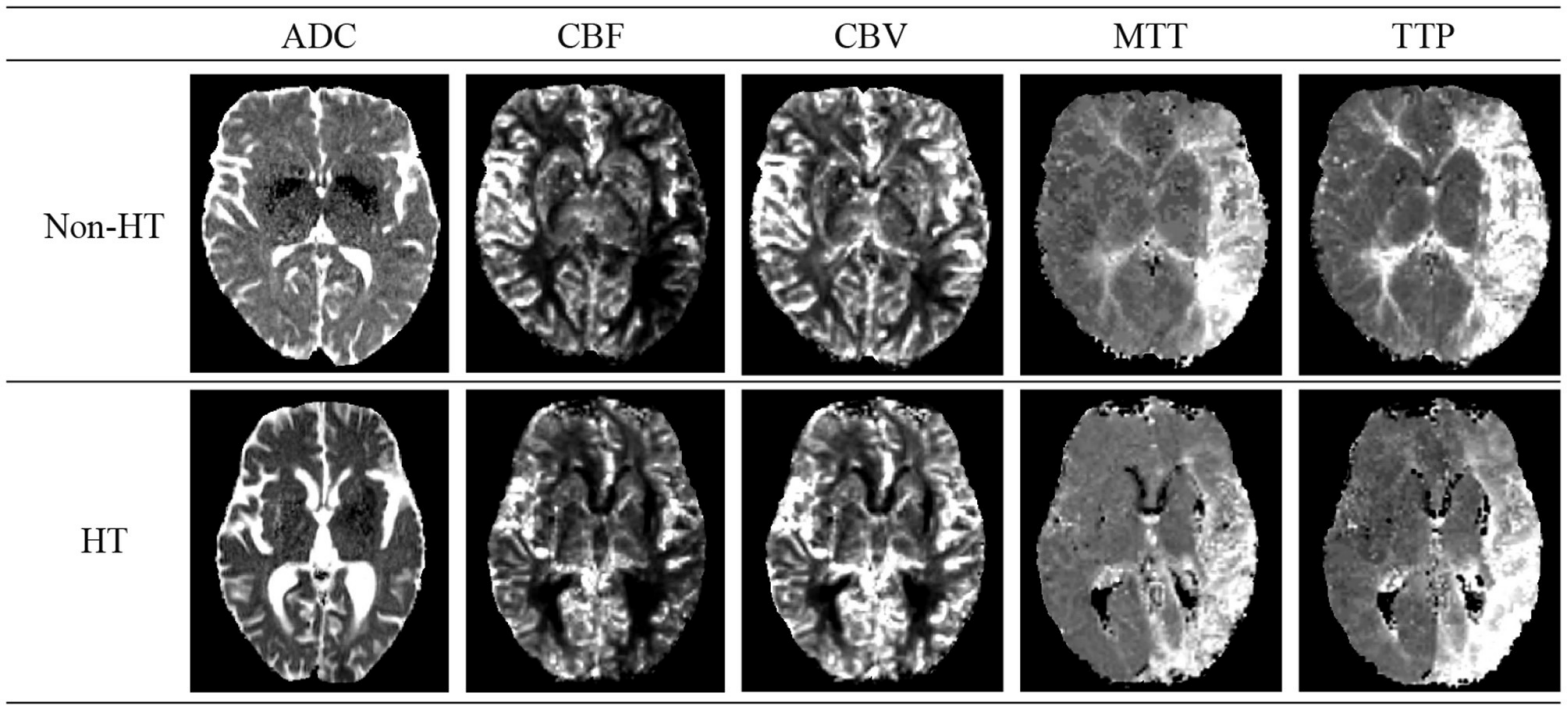
Schematic diagrams of cerebral Hemorrhagic Transformation (HT) and non-HT in stroke patients. The details of five modals can be found in Section 2.1.

Acute Ischemic Stroke (AIS), the second leading cause of global mortality, can lead to HT—a condition where initially absent bleeding in cranial scans appears in later examinations, significantly raising disability and mortality rates. Figure 2 shows schematic diagrams illustrating HT in patients with AIS, comparing cases with HT and Non-HT. The use of intravenous rt-PA thrombolysis, a key treatment for AIS, increases HT risks, complicating the decision-making process for clinicians regarding its administration. This uncertainty can hinder patient recovery and limit the effectiveness of thrombolytic therapy. Thus, early identification of patients at risk of developing HT post-AIS is crucial for guiding therapeutic decisions and optimizing patient outcomes, highlighting the importance of research and development in predictive diagnostics (Vidal et al., 2013; Paciaroni et al., 2018).

Early attempts to resolve challenges in brain MRI classification and HT prediction primarily utilized statistical analyses of clinical data or employed radiomics, leveraging machine learning for feature extraction and classification (Mazya et al., 2012; Chen et al., 2021).

These methods, however, suffered from limited accuracy. The advent of deep learning in 2012 marked a significant shift (LeCun et al., 2015), with numerous deep learning-based approaches being introduced for enhanced classification of brain MRI images (Nielsen et al., 2018). Specifically, Convolutional Neural Networks (CNNs) have been widely adopted for brain tumor classification, demonstrating notable success across various datasets (Seetha and Raja, 2018; Deepak and Ameer, 2019). For example, Seetha and Raja (2018) achieved a classification accuracy of 97.5% in differentiating tumor from non-tumor regions, while Deepak and Ameer (2019) reported a 98% accuracy in classifying gliomas, meningiomas, and pituitary tumors using GoogLeNet pre-trained on ImageNet (Deng et al., 2009). In the context of HT following AIS, CNNs have also shown promise in predicting tissue prognosis and identifying penumbral tissue, with Jiang et al. (2023) developing a model in 2021 that predicts HT post-thrombectomy with significant accuracy.

The Attention mechanism, originating in visual imagery and computer vision, emulates human attention by focusing on critical details and ignoring irrelevant information (Vaswani et al., 2017). It has been increasingly integrated with deep learning, employing masks to highlight essential features in images and enable networks to identify areas of interest. Recently, the integration of Attention mechanisms with deep learning has been explored in medical imaging tasks, enhancing feature detection and segmentation capabilities (Gou et al., 2020; Zhang et al., 2021). For instance, Sinha and Dolz (2020) introduced self-attention mechanisms to improve contextual dependency capture, yielding superior performance in medical image segmentation by enhancing relevant features and suppressing noise.

Integrating features across multiple scales is crucial for enhancing segmentation accuracy. Modern detection and segmentation networks leverage CNNs for hierarchical feature extraction, transitioning from low-level, detailed features to high-level, semantic features as network depth increases. While low-level features are rich in detail and position information, they suffer from low semantic content and noise due to minimal convolutional processing. High-level features, in contrast, contain valuable semantic information but lack resolution and detail perception. Recent research has aimed at improving segmentation and classification by merging features from various scales (Lin et al., 2017).

To address these challenges, we have introduced a novel classification technique that leverages multi-scale feature fusion and attention mechanisms for Multi-modal brain disease classification tasks, termed *M*^3^. Unlike conventional approaches, *M*^3^ begins by segmenting the original images and their associated disease-related region labels, utilizing a segmentation network that integrates multi-scale features with attention mechanisms to precisely extract features from regions associated with the disease. Subsequently, Principal Component Analysis (PCA) (Wold et al., 1987) is applied to refine these features, isolating those with the greatest discriminatory power. These selected features are then classified using a Support Vector Machine (SVM) classifier (Chang and Lin, 2011), culminating in the final classification outcomes. This method has undergone rigorous validation across Multi-modal brain tumor and AIS datasets, demonstrating robust classification capabilities in both contexts. Furthermore, extensive ablation studies have further confirmed the effectiveness of the proposed modules, highlighting their contribution to enhanced classification performance.

## 2 Materials and methods

### 2.1 Datasets

#### 2.1.1 Dataset 1

Table 1 shows the multicenter trial data of brain tumor used in this study. The magnetic resonance imaging (MRI) data of gliomas and solitary brain metastases from Department 1 and Department 2 were obtained from the Linyi People's Hospital Affiliated to Shandong Second Medical University. The MRI data of brain metastases from Department 1 include 87 patients with solitary brain metastases (48 males, 39 females, mean age 58.4 ± 11.2 years), which were collected from GE (23), SIEMENS (35), and Philips 3T MRI systems (29). All patients underwent MRI imaging with T1 contrast-enhanced modal (T1ce), T2, and FLAIR (Fluid Attenuated Inversion Recovery) modals. The MRI data of gliomas and brain metastases from Department 2 were also acquired from the GE 3T MRI system, comprising 88 brain tumor patients, including 45 cases of solitary brain metastases (32 males, 13 females, mean age 57.1 ± 10.2 years) and 43 cases of gliomas (25 males, 18 females, mean age 53.0 ± 11.7 years). MRI images of all patients included T1ce, T2, and FLAIR modals. The MRI data from Department 1 and 2 were annotated by three clinical doctors, each with over ten years of experience and holding intermediate or senior professional titles to identify tumor regions. Considering the limited quantity of glioma data, we also utilized glioma MRI data publicly available from the BraTS 2020 Challenge. This challenge provided data with T1, T1ce, T2, and FLAIR modals, along with segmentation labels of tumor regions. To align with our private data, we only used T1ce, T2, and FLAIR modals.

**Table 1:** Patient information for glioma and metastasis, including source, number of patients, age distribution, and utilized modals.

**Category**	**Source**	**# Cases**	**Age**	**Modals**
Metastases	Department 1	87	58.4 ± 11.2	T1ce, T2, FLAIR
	Department 2	45	57.1 ± 10.2	
Gliomas	Department 2	43	53.0 ± 11.7	
	BraTS2020	369	61.2 ± 11.8	

#### 2.1.2 Dataset 2

Table 2 shows the trail data of AIS used in this study. We selected a total of 136 patients with AIS admitted to the Linyi People's Hospital Affiliated to Shandong Second Medical University from February 2016 to September 2018 as the dataset for stroke. After screening for inclusion, 71 cases met the criteria for the trial, including 11 cases with HT and 60 cases with None-HT. Both the initial and follow-up MRI images were acquired using an 8-channel head coil on a Siemens 3.0T MR imaging system (MAGNETOM Verio; Siemens Medical Solutions, Germany). The initial examination modals included T1w, DWI, PWI, and SWI modals for clear detection of hemorrhage, or CT examination. No intracranial bleeding was detected in the initial examination of all cases. All patients received intravenous thrombolysis treatment and underwent follow-up examinations within 1–3 days. After acquisition at the scanning workstation, the Siemens MR Syngo medical image post-processing workstation automatically generated images of ADC, SWI_MIP, CBF, CBV, MTT, TTP, and other parameter modals based on DWI, SWI, and PWI images. We selected ADC (Apparent diffusion coefficient), CBF (Cerebral blood flow), CBV (Cerebral blood volume), MTT (Mean transit time), and TTP (Time to peak) as the five modals for inclusion in the study.

**Table 2:** Stroke patient information, including source, number of patients, age distribution, and utilized modals.

**Category**	**Source**	**# Cases**	**Age**	**Modals**
HT	Department 3	11	64.00 ± 13.20	ADC, CBF, CBV, MTT, TTP
None-HT		60	62.07 ± 11.24	

#### 2.1.3 Delineation protocols

The data annotations were conducted by three senior clinical experts, each with over ten years of experience. Unlike annotations performed by junior or general practitioners, the annotations by these highly experienced experts are more reliable and accurate.

The experts followed a specific protocol for delineation: (1) Each expert independently annotated three different parts of the data. (2) The annotations were then reviewed by the other two experts. (3) For data where there were significant differences in opinion, the three experts discussed the discrepancies together and made a decision based on the majority rule. (4) All annotated data were finalized only after achieving consensus among the three experts.

The software used for creating the tumor delineations was ITK-SNAP (version 3.8), available from the official website (http://www.itksnap.org).

### 2.2 Methodology

**Figure 3:**
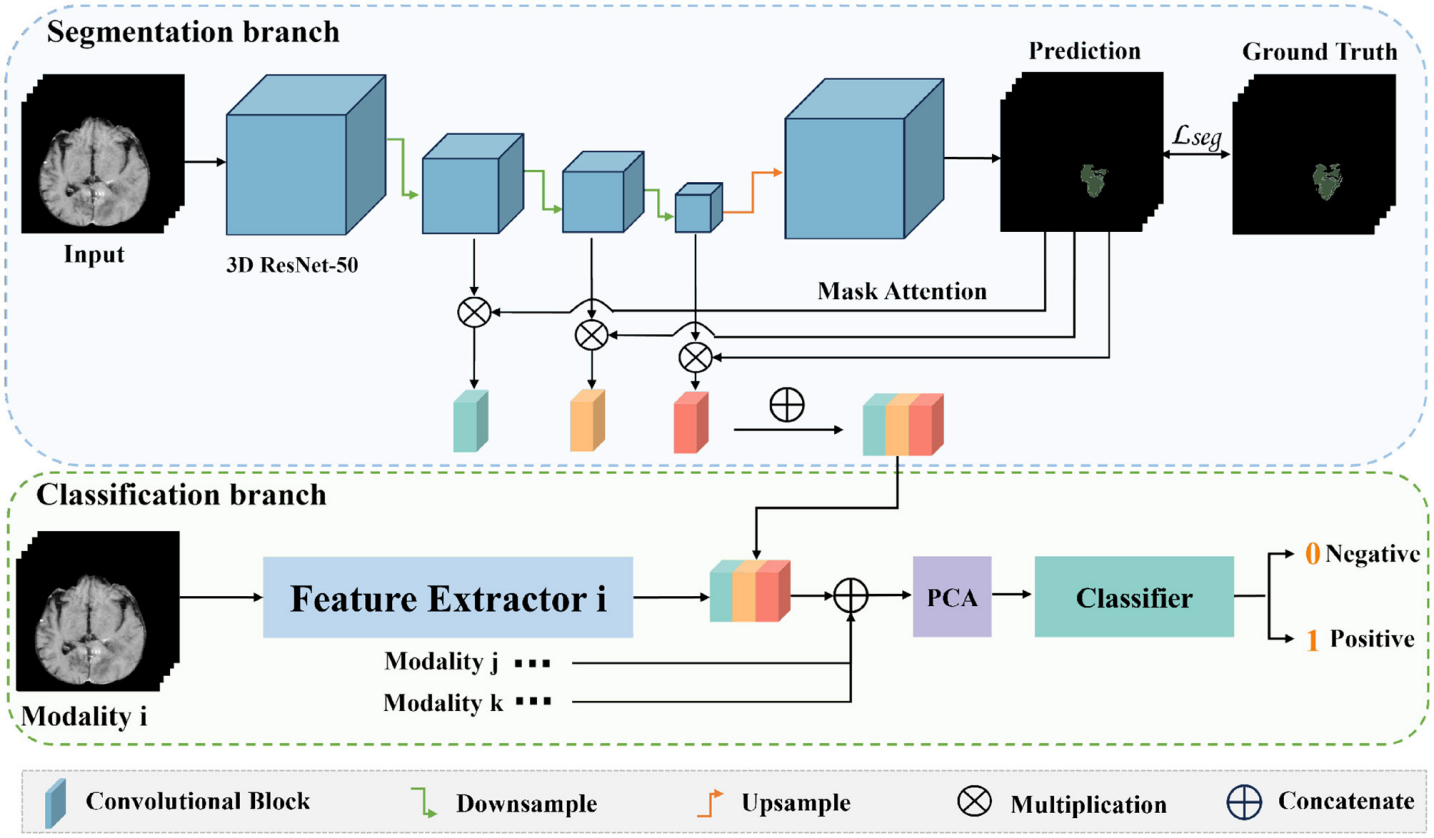
The overall framework of *M*^3^. Firstly, we train a segmentation model for the tumor areas or abnormal areas related to bleeding transformation marked by our doctors. After the segmentation network is trained, we multiply the probability prediction map with the features extracted from downsampling to form an attention mechanism to eliminate interference from irrelevant area features. Then, we concatenate the three layers of features extracted from downsampling, fuse multi-scale features, use PCA for feature dimensionality reduction, and finally use SVM for classification.

The entire network framework of *M*^3^ is depicted in Figure 3. Initially, we trained a segmentation network with the input being preprocessed original images from each modal and their corresponding segmentation labels. These segmentation labels, which are manually delineated by doctors, correspond to regions that are highly relevant to the disease or are tumor areas. During the training of the segmentation network, the network's output is multiplied with the feature maps from each layer in the downsampling process. Subsequently, the feature maps obtained after three downsampling operations are concatenated to integrate multi-scale features for classification. After training the segmentation model, we transferred the weights of the segmentation network's encoder for extracting high-level semantic features for classification. Once the feature extraction is completed, we reduced the dimensionality of the extracted features using PCA. We then utilized a SVM for classification prediction to obtain the classification results for each modal. Additionally, we conducted a control experiment for multi-modal fusion, combining the results from multiple modals for classification, and compared their predictive performance.

#### 2.2.1 Preprocessing

For brain tumor and stroke data, we conducted meticulous image preprocessing. Each brain tumor patient's T1ce, T2, FLAIR images, and AIS patient's T1WI, DWI, and PWI images were saved in DICOM format, which can be converted to NIFTI format using MRICron software.

For private data from departments 1 and 2, the specific processing steps are as follows:

- We used ANTs-N4 (Tustison et al., 2010) for bias field correction on all images.
- FSL-FLIRT (Smith et al., 2004) was employed to register the multimodal data of each patient, aligning T2 and FLAIR data to the T1ce images.
- FSL-BET (Smith et al., 2004) was applied to remove non-brain tissues from the T2 images of each patient, using a threshold of 0.5, and obtaining a brain tissue mask for each patient. This mask was then used to remove non-brain tissues from other modalities.

Since the data is from multiple centers, all images underwent intensity normalization, standardizing the grayscale histograms of all images from each modal. It should be noted that for the glioma MRI provided by BraTS2020, which has already undergone preprocessing such as skull stripping and cross-modal registration, we only performed normalization on its grayscale histograms.

For AIS images, we first used ANTs-N4 (Tustison et al., 2010) software for bias field correction and performed grayscale histogram normalization. Subsequently, we used FSL (Smith et al., 2004) software to align the DWI (*b* = 0) and PWI images to the T1WI image space, and applied the transformation matrices generated to the DWI (*b* = 1,000) images (and their associated ADC images) and PWI images (and their associated CBF, CBV, MTT, and TTP images) to ensure alignment with the T1WI space. Finally, we used FreeSurfer (Fischl, 2012) software to automatically segment each patient's T1WI images based on a brain template, obtaining brain region labels and brain tissue masks, and used the obtained masks to remove non-brain tissues from the images.

#### 2.2.2 Feature extraction

We first train a segmentation network by inputting the original images along with their corresponding annotations. Once training is complete, we transfer the weights of the encoder to another network for extracting classification features. During this process, we multiply the probability maps obtained after training with each layer's feature maps to form an attention mechanism. Then, we concatenate the feature maps obtained after three times of downsampling to fuse multi-scale features for classification. The process of feature extraction is depicted in Equation (1).


(1) $$\begin{array}{c} F=\overset{N}{\underset{i=1}{\sum }}\left(D\left(E_{N}\left(x\right)\right)◦E_{i}\left(x\right)\right), \end{array}$$

where *x* represents the input image, *F* represents the final output features, *E* represents the encoding operation, *D* represents the decoding operation, *N* represents the number of down-sampling operations, and *i* = 1, …, *N* represents the *i*-th down-sampling operation.

#### 2.2.3 Segmentation network architecture

We trained a segmentation network using the regions annotated by doctors as ground truth. Subsequently, we transferred the weights of the encoder of the segmentation network to a feature extraction network for classification. The overall structure of the segmentation network is based on MED3D (Chen et al., 2019), adopting a conventional encoder-decoder architecture. We initialized the weights with pretrained weights from MED3D (Chen et al., 2019). The encoder adopts the 3D ResNet-50 (He et al., 2016), known for its excellent feature representation capability. The decoder consists only of an upsampling layer to restore the size of the feature maps to the original size, facilitating the training of the segmentation network using segmentation masks. As described in MED3D, concentrating the network's feature representation capability in the encoder is advantageous for subsequent transfer to downstream classification tasks.

The training details of the entire segmentation network are as follows: ResNet-50 accepts images of the original size of 155 × 280 × 280 (length × width × height), and after undergoing convolution and max pooling operations by ResNet-50, they are transformed to a size of 39 × 70 × 70 with 256 channels. The images then become 20 × 35 × 35 (length × width × height), with the number of channels gradually increasing to 256, 1,024, and 2,048. Ultimately, the size of the feature maps from the encoder is 2,048 × 20 × 35 × 35 (number of channels × length × width × height). Subsequently, through an upsampling operation, the feature maps are restored to 39 × 70 × 70, with 2 channels, to obtain a predicted segmentation result. At this stage, to ensure that the dimensions of the ground truth and the prediction match for the convenience of loss calculation, the ground truth must be cropped to correspond to the size of the prediction, making their sizes uniform. The calculated loss is then fed back into the network to update the parameters, with a total of 200 iterations performed until the network converges.

During the training of the segmentation network, we mixed the two types of MRI images and used only their segmentation labels to train the network. Additionally, no category label information was used during the training of the segmentation network. Thus, during the training process, the network was unaware of whether the images corresponded to gliomas or solitary brain metastases, or whether they were related to bleeding or non-bleeding conditions.

Once the segmentation network is trained, we input the MRI images into the network to extract the highest-level semantic features. Given that this network is adept at segmenting the abnormal or tumor regions, the high-level semantic features extracted are pertinent to these areas. By employing these features for subsequent classification tasks, we can minimize the interference from irrelevant regional features.

### 2.3 Attention mechanism

The principle of the attention mechanism lies in identifying key features in image data through a layer of new weights. Through learning and training, deep neural networks can learn to focus on regions of interest in any given new image. In brain slice images, tumor areas or abnormal regions related to bleeding only occupy a small part of the image, and information from other areas may interfere with subsequent feature extraction. Therefore, we introduce the attention mechanism to allow the network to automatically focus on abnormal regions that are helpful for classification while ignoring normal areas.

The specific approach is shown in the Figure 3: the original image is input into the well-trained network, which outputs a probability map and a feature map. As shown in Equation (1), when we input the original image *x* into the trained network, it outputs probability maps *D*(*E*_*N*_(*x*)) and feature maps *E*_*i*_, (*i* = 1, …, *N*). The probability map reflects the probability of each point on the image being an abnormal region. By multiplying the probability map with the feature maps of the downsampling layers, we can make the network pay more attention to the features of abnormal regions during feature extraction, reducing interference from normal areas.

### 2.4 Multi-scale feature fusion

The ResNet-50 network (He et al., 2016) we employed extracts features of abnormal ROI regions in a hierarchical manner. During the downsampling process of images, the low-level features obtained by this network have higher resolution, containing more positional and detailed information. However, due to fewer convolutional operations, these features have lower semantic meaning and are more susceptible to noise interference. In contrast, high-level features possess stronger semantic information but lower resolution, resulting in poorer perception of details. Therefore, in deep learning, the fusion of these features proves significantly beneficial for both detection and segmentation tasks.

### 2.5 Feature dimensionality reduction and classification

After attention mechanism and multi-scale feature fusion, we extracted 7,168-dimensional features from each region. Due to the high dimensionality of the features, there exists redundancy among them, which may lead to overfitting. Therefore, it is necessary to reduce the dimensionality of the features to eliminate their correlation. Here, we employed PCA for dimensionality reduction, selecting the most discriminative feature set related to hemorrhage transformation. The reduced-dimensional features were then used for classification prediction using SVM. When performing classification prediction with SVM, we used classification labels, meaning that the features extracted by the segmentation network were identified as being from patients with glioma or brain metastasis, or from patients with HT or non-HT. For both classification tasks, we first computed the prediction results for each modal separately, and then fused the features from multiple modals for prediction.

### 2.6 Statistical analysis

We conducted statistical analysis on the results obtained from each modality, comparing the area under the ROC curve (AUC) of various prediction models and calculating the F1 score (F1) and accuracy (ACC). A 5-fold cross-validation was employed to optimize the results. The statistical analysis was carried out using the R language in conjunction with the PyTorch package (Paszke et al., 2019). Given the extensive number of experiments and comparisons involved, we decided against listing each individual *p*-value to maintain readability and conciseness. Nonetheless, all the metrics reported (F1, ACC) in Tables 3–**5** were rigorously tested, and each showed *p*-values < 0.05, demonstrating statistically significant differences.

**Table 3:** Performance metrics of *M*^3^ for brain tumor classification, detailing F1 and ACC results for individual MRI modals and multi-modal fusion.

	**F1**	**ACC**
T1ce	0.9264 ± 0.05	0.9463 ± 0.02
T2	0.9163 ± 0.05	0.9382 ± 0.02
FLAIR	0.8567 ± 0.07	0.8964 ± 0.01
Multimodal	0.9405 ± 0.03	0.9554 ± 0.01

## 3 Results

### 3.1 Experiment setup

The experimental development environment is as follows: programming language Python 3, deep learning framework PyTorch, processor Intel(R) Core(TM) i5-9600K CPU @ 3.70 GHz, display adapter NVIDIA GeForce RTX 2080 Ti, operating system Ubuntu 18.04, and medical image data reading and writing operations provided by SimpleITK (Yaniv et al., 2018).

### 3.2 Performance of classification prediction

**Figure 4:**
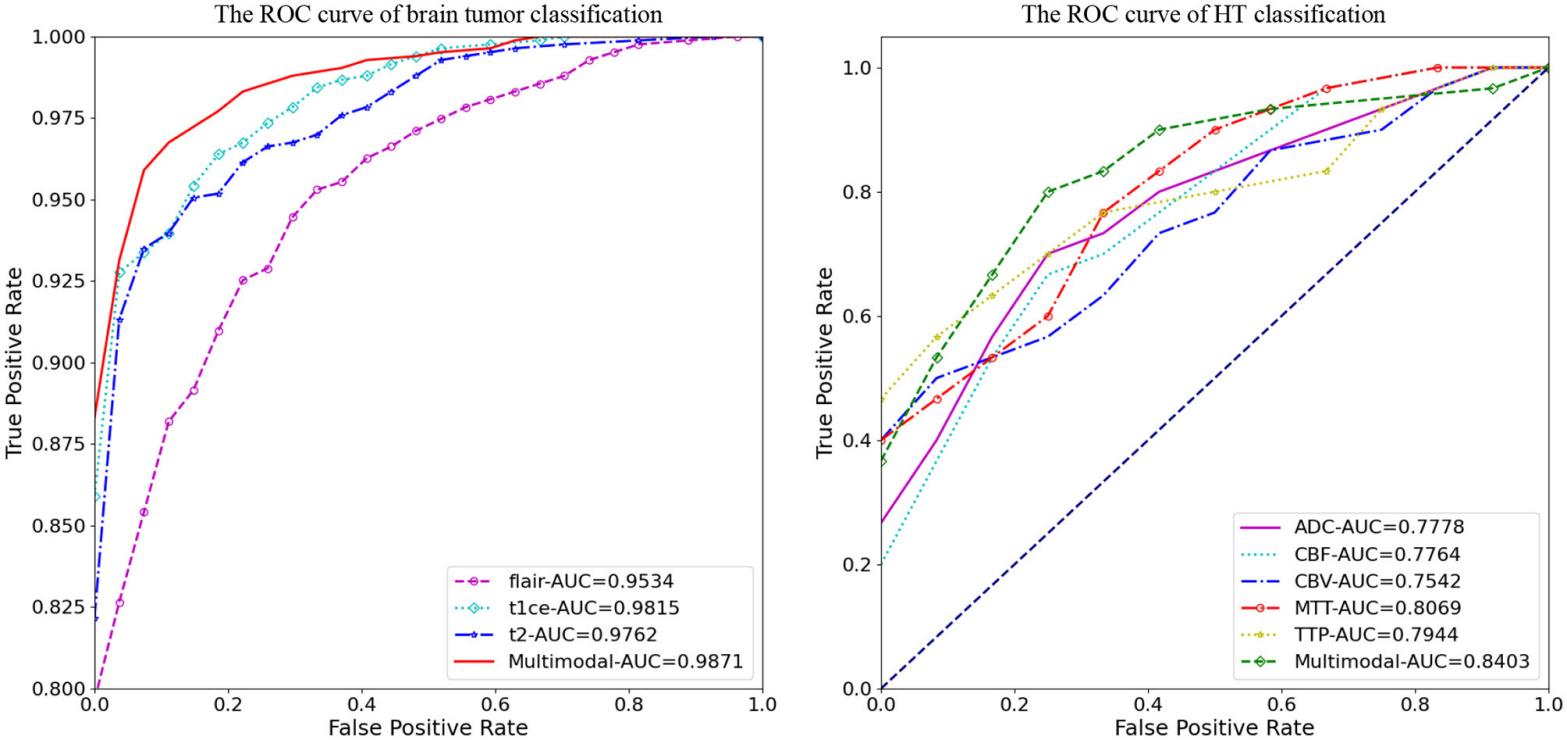
Comparative analysis of AUC results for *M*^3^, displaying performance on individual modals and the enhanced outcomes from multi-modal fusion.

Figure 4 illustrates the classification prediction performance for brain tumors and cerebral hemorrhage transformation in stroke. From the ROC curves, it can be observed that the classification prediction results vary for different modalities or modals of images. For the brain tumor classification task, the classification performance of the three single-modal images all exceeds 0.95, with the best performance achieved by the t1ce modal at 0.9815 (light blue line), while the FLAIR modal has the lowest classification performance at 0.9543. As for the cerebral hemorrhage transformation classification prediction task, the classification prediction performance of the five single-modal images all exceeds 0.75, with the MTT modal showing the best performance at 0.8069 and CBV demonstrating the lowest performance at 0.7542. For both tasks, we conducted multimodal fusion experiments. The AUC value for brain tumor fusion of the three modalities is 0.9871, while for cerebral hemorrhage transformation prediction, it is 0.8403. The results indicate that the experimental results of multimodal fusion are higher than those of single modal, demonstrating the effectiveness of multimodal fusion.

Tables 3, 4 display the F1 and ACC for the classification prediction of the two tasks. It can be observed that the results of F1 and ACC are similar to those of AUC. For brain tumor classification prediction, the F1 and ACC values for each modal all exceed 0.85. The t1ce modal demonstrates the best classification performance, with F1 and ACC reaching 0.9246 and 0.9463, respectively, while the FLAIR modal shows the lowest performance at 0.8567 and 0.8964. As for cerebral hemorrhage transformation prediction, TTP exhibits the best performance, with F1 and ACC reaching 0.7905 and 0.8457, respectively, while the FLAIR modal shows the lowest performance. Similarly, the performance of multimodal fusion is superior to that of single modal. For brain tumor multimodal classification prediction, the F1 and ACC values reach 0.9405 and 0.9554, respectively, outperforming the results of single modal. The multimodal results of cerebral hemorrhage transformation prediction also outperform those of single modal, with F1 and ACC values reaching 0.7943 and 0.8533 for multimodal fusion.

**Table 4:** Performance metrics of *M*^3^ for HT classification, detailing F1 and ACC results for individual MRI modals and multi-modal fusion.

	**F1**	**ACC**
ADC	0.7428 ± 0.11	0.8066 ± 0.06
CBF	0.7093 ± 0.15	0.7400 ± 0.14
CBV	0.7702 ± 0.13	0.8400 ± 0.08
MTT	0.7383 ± 0.15	0.7800 ± 0.13
TTP	0.7905 ± 0.12	0.8457 ± 0.05
Multimodal	0.7943 ± 0.10	0.8533 ± 0.04

### 3.3 Ablation study

**Figure 5:**
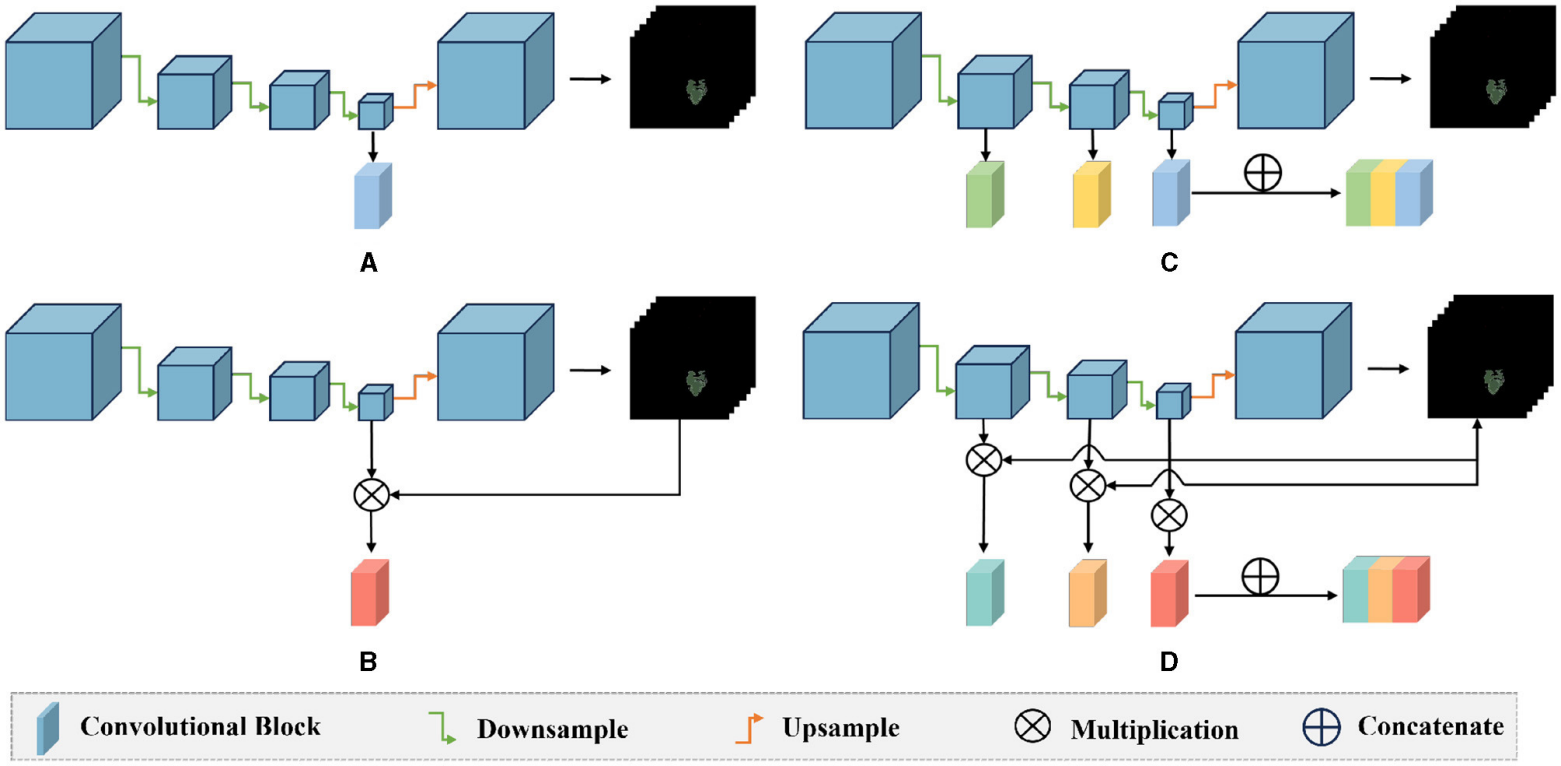
Comparison diagram of the ablation methods and our method: **(A)** deep learning: only the last layer features of the encoder are used for classification prediction; **(B)** mask attention: the probability map is multiplied with the features of the last layer of the encoder to exclude the interference of irrelevant features; **(C)** multiscale: the three layers of features extracted by the encoder's down-sampling are concatenated, and multi-scale features are used for classification; **(D)** our method: the probability map is multiplied with the three layers of features extracted by the encoder, and then these three layers of features are concatenated for classification.

**Figure 6:**
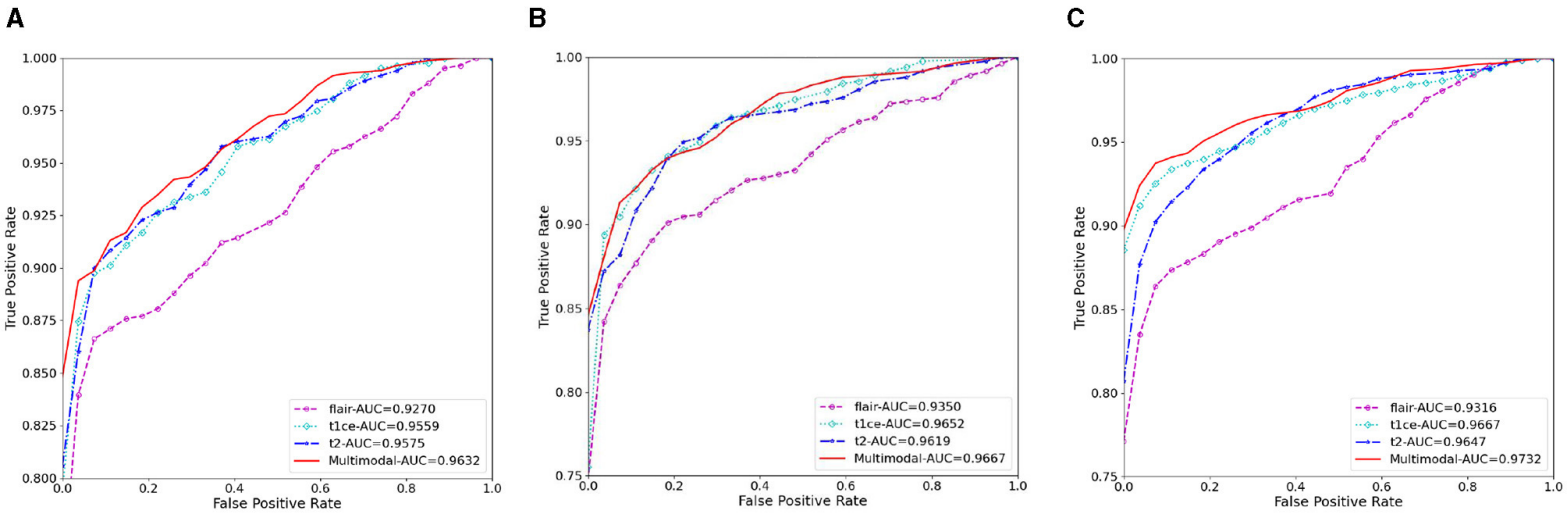
The AUC results of three ablation methods for brain tumor classification. **(A)** Deep learning; **(B)** mask attention; **(C)** multiscale.

**Figure 7:**
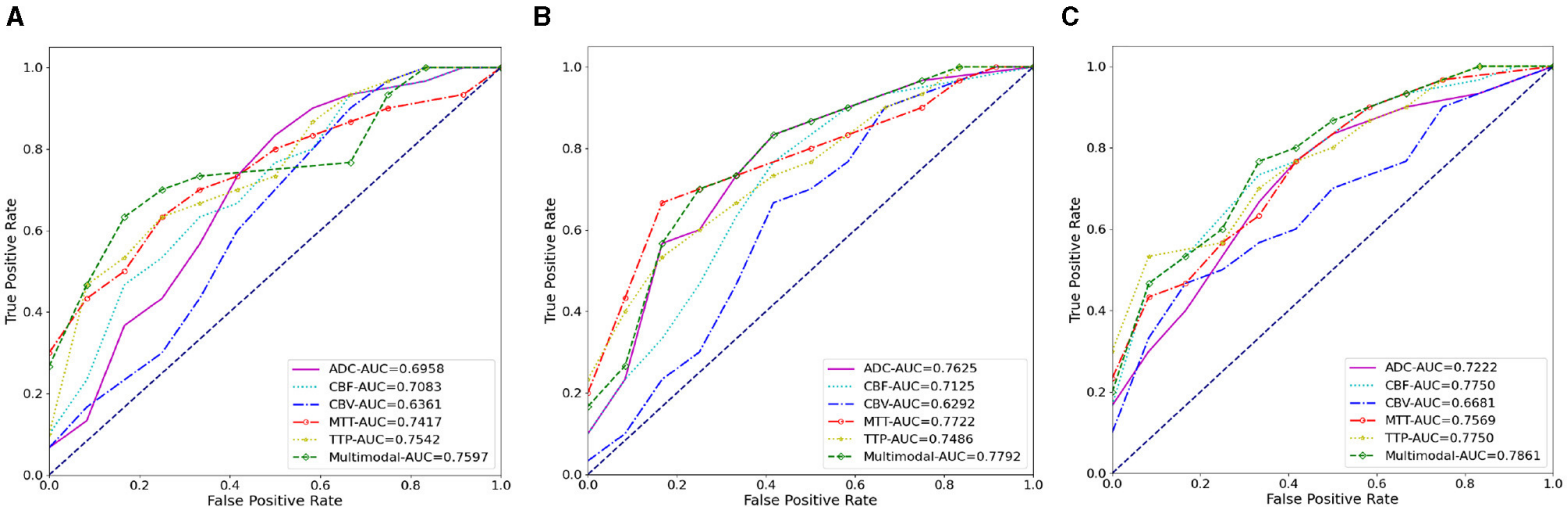
The AUC results of three ablation methods for HT prediction classification. **(A)** Deep learning; **(B)** mask attention; **(C)** multiscale.

We also conducted ablation experiments to demonstrate the effectiveness of our proposed attention mechanism and multi-scale feature fusion. In the ablation study, we used the same data as the main experiment, along with the identical data partitioning. The difference lies in the modifications made to the network architecture. We compared three methods: pure deep learning (Deep Learning), deep learning with only the attention mechanism added (Mask Attention), and deep learning with only multi-scale feature fusion added (Multiscale). The details of three methods are shown in Figure 5. The ROC curve is shown in Figures 6, 7, The F1 and ACC is listed in Tables 5, 6. From the results of AUC, F1, and ACC, it can be observed that the results of adding either the attention mechanism or multi-scale feature fusion are better than pure deep learning. However, all three methods perform worse than *M*^3^ (which can be seen in Tables 3, 4), confirming the effectiveness of our proposed approach.

**Table 5:** The ablation study results of *M*^3^ for brain tumor classification.

	**Deep learning**		**Mask attention**		**Multiscale**	
	**F1**	**ACC**	**F1**	**ACC**	**F1**	**ACC**
T1ce	0.8896 ± 0.05	0.9136 ± 0.01	0.9005 ± 0.06	0.9254 ± 0.06	0.9114 ± 0.05	0.9318 ± 0.02
T2	0.8867 ± 0.06	0.9136 ± 0.01	0.8887 ± 0.05	0.9182 ± 0.02	0.8938 ± 0.05	0.9190 ± 0.01
FLAIR	0.8627 ± 0.06	0.8873 ± 0.02	0.8595 ± 0.06	0.8900 ± 0.01	0.8616 ± 0.06	0.8873 ± 0.01
Multimodal	0.8946 ± 0.05	0.9172 ± 0.02	0.9081 ± 0.04	0.9291 ± 0.01	0.9250 ± 0.05	0.9427 ± 0.02

**Table 6:** The ablation study results of *M*^3^ for HT predict classification.

	**Deep learning**		**Mask attention**		**Multiscale**	
	**F1**	**ACC**	**F1**	**ACC**	**F1**	**ACC**
ADC	0.6699 ± 0.11	0.7333 ± 0.12	0.6719 ± 0.15	0.7133 ± 0.14	0.6817 ± 0.12	0.7333 ± 0.10
CBF	0.7090 ± 0.12	0.7533 ± 0.14	0.7285 ± 0.15	0.7733 ± 0.13	0.7092 ± 0.12	0.7533 ± 0.11
CBV	0.6034 ± 0.11	0.6600 ± 0.12	0.6714 ± 0.14	0.7400 ± 0.13	0.6071 ± 0.11	0.6600 ± 0.11
MTT	0.7408 ± 0.12	0.8133 ± 0.11	0.6969 ± 0.14	0.7466 ± 0.13	0.7473 ± 0.13	0.7600 ± 0.13
TTP	0.6861 ± 0.14	0.7400 ± 0.14	0.7657 ± 0.13	0.7800 ± 0.09	0.6811 ± 0.14	0.7333 ± 0.14
Multimodal	0.7530 ± 0.13	0.7550 ± 0.12	0.7666 ± 0.12	0.7866 ± 0.10	0.7405 ± 0.12	0.7655 ± 0.11

Moreover, we expanded our analysis by using Dataset 2 to test more advanced feature transformation and classification methods, including direct use of complex CNNs for classification, as well as replacing SVM with Random Forest (RF), Gradient Boosting Machines (GBM), and a three-layer nonlinear Multilayer Perceptron (MLP).

The results of these tests are presented in Table 7. Here is a summary of the findings:

**Table 7:** The ablation study results of feature transformation and classification methods.

**Multimodal**	**F1**	**ACC**
Ours	0.794 ± 0.10	0.853 ± 0.04
Direct CNN	0.720 ± 0.15	0.818 ± 0.07
Ours-RF	0.787 ± 0.09	0.839 ± 0.04
Ours-GBM	0.790 ± 0.11	0.841 ± 0.05
Ours-MLP	0.791 ± 0.13	0.841 ± 0.05

(1) The direct use of CNNs for simultaneous segmentation and classification (Direct CNN) showed clear disadvantages compared to our method where features are first extracted from the segmentation ROI, followed by classification. This is likely due to limited data size leading to inadequate feature extraction and significant overfitting.

(2) The performance of RF, GBM, and MLP was comparable to SVM, with no significant differences in performance (*p*-values > 0.05). This comprehensive testing underscores that when the initial feature extraction is robust, the choice among these advanced classifiers does not significantly impact performance.

These results confirm the appropriateness of our methodological choices given the constraints and characteristics of our data.

## 4 Discussions

The health of the brain is crucial for overall wellbeing, with brain tumors and strokes being two common conditions that can cause severe damage to the brain. Currently, there is a lack of effective methods in clinical practice for accurately classifying brain gliomas and brain metastases, as well as predicting HT in strokes.

Previous approaches (Aerts et al., 2014; Chen et al., 2021) have relied on clinical information and radiomics for feature extraction, but their classification accuracy has been limited. Taking the prediction of HT as an example, we conducted a preliminary experiment. If we only used clinical information for classification, the AUC value was only around 0.6. If we used radiomic methods to extract features for prediction, the highest classification AUC did not exceed 0.75, proving that traditional methods are not adequate for this task. The introduction of deep learning has alleviated some of these issues, but accuracy remains a concern. Building upon existing deep learning methods, we incorporated attention mechanisms and multi-scale feature fusion to allow the network to focus more on the features of ROI and integrate features from both deep and shallow layers for classification. *M*^3^ achieves high-precision classification of brain tumors and predicts whether hemorrhage occurs after thrombolytic therapy in stroke patients.

In recent years, many methods (Nielsen et al., 2018; Jiang et al., 2023) have been proposed to address the classification of brain images. Previously, most relied on clinical information for classification, but clinical scoring mechanisms are subject to individual bias, and doctors must have a clear understanding of the patient's condition, leading to unreliable classifications (Mazya et al., 2012; Strbian et al., 2014). With the development of radiomics, researchers have begun using radiomics to extract features and then classify them using machine learning algorithms (Aerts et al., 2014). However, radiomics only captures shallow features such as grayscale and texture. Although deep learning has improved classification accuracy by extracting deep features, previous methods often only used simple downsampling to extract features. However, features from non-ROI regions in images may interfere with the network, and as the network downsamples, shallow features are gradually lost. Therefore, we introduced mask attention mechanisms and multi-scale feature fusion to allow the network to focus more on ROI and integrate features from multiple scales, resulting in more favorable feature extraction for classification.

After feature extraction, due to the high dimensionality and redundancy of features, we used PCA for dimensionality reduction, followed by inputting the reduced features into SVM for classification. Firstly, the decision to employ linear methods like PCA for feature transformation and SVM for classification was grounded on the high-quality features extracted from our deep-learning segmentation framework, which is based on multi-scale and mask-attention mechanisms. These features effectively encapsulate the critical characteristics of ROI essential for downstream tasks, providing a robust foundation for the effective application of linear methods. Secondly, the size of the clinical datasets used (87 + 88 patients in Dataset 1 and 71 patients in Dataset 2) poses challenges. Given the relatively small scale of these datasets, deploying complex nonlinear models such as deep neural networks could lead to overfitting. In contrast, simpler linear methods entail lower risks of overfitting and are thus more suitable under these conditions.

Assessing the quality of delineations through inter- and intra-rater variability analysis is important in this paper. Firstly, we would like to emphasize that all our data annotations were conducted by three senior clinical experts, each with over ten years of experience and holding intermediate or senior professional titles. Unlike annotations performed by junior or general practitioners, the annotations by these experienced experts are highly reliable and accurate. Furthermore, each expert's independent annotations were rigorously reviewed by the other two experts, and all annotated data were finalized only after achieving consensus among the three experts. Therefore, the inter-rater and intra-rater variability of our data annotations is highly reliable.

We have conducted additional analysis on Dataset 2 using 71 cases to assess both inter-rater and intra-rater variability. For the inter-rater variability analysis, we randomized the annotations such that each expert annotated cases they had not previously annotated, following the same protocol of review and consensus by the other two experts. We retrained the multimodal model using this new set of annotations and found no significant difference (*p*-value < 0.05) between the predictions of this model and the model reported in the manuscript. This indicates that the inter-rater variability of our dataset annotations is stable and reliable. For the intra-rater variability analysis, each expert re-annotated the data following the same protocol of review and consensus by the other two experts. We retrained the multimodal model with this new set of annotations and again found no significant difference (*p*-value < 0.05) between the predictions of this model and the model reported in the manuscript. This demonstrates that the intra-rater variability of our dataset annotations is also stable and reliable.

Our proposed method has some limitations. Firstly, as a data-driven deep learning task, the two datasets we used are relatively limited. Secondly, the interpretability of the features extracted by deep learning is weak. Although the effectiveness of the two modules we proposed has been verified in classification results, their effectiveness lacks theoretical proof.

In the future, we will validate our model using multi-center datasets, and we will also compare more deep learning feature extraction methods to further improve our proposed attention mechanisms and multi-scale feature fusion.

## 5 Conclusions

We have proposed a novel method called *M*^3^ to address the classification problem of multi-modal brain diseases, which integrates mask attention and multi-scale feature fusion module. The mask attention module allows the network to focus on the regions regions that are highly relevant to the disease, while the multi-scale feature fusion module combines features from multiple scales. *M*^3^ has been validated on brain tumor data and brain hemorrhage conversion data, and ablation experiments have fully demonstrated the effectiveness of the two modules we proposed.

## Data availability statement

The data analyzed in this study is subject to the following licenses/restrictions: although the dataset used cannot be directly made public, it can be obtained through legitimate requests with valid reasons. Requests to access these datasets should be directed to DQ, yiyuangaigeban@163.com.

## Ethics statement

The studies involving humans were approved by Linyi People's Hospital, Shandong, China. The studies were conducted in accordance with the local legislation and institutional requirements. The human samples used in this study were acquired from primarily isolated as part of your previous study for which ethical approval was obtained. Written informed consent for participation was not required from the participants or the participants' legal guardians/next of kin in accordance with the national legislation and institutional requirements.

## Author contributions

DQ: Conceptualization, Data curation, Funding acquisition, Project administration, Resources, Supervision, Writing – original draft, Writing – review & editing. GK: Conceptualization, Data curation, Methodology, Writing – original draft, Writing – review & editing. CW: Data curation, Formal analysis, Investigation, Methodology, Writing – original draft, Writing – review & editing. ZZ: Data curation, Resources, Validation, Writing – review & editing. CY: Data curation, Validation, Visualization, Writing – review & editing.

## References

[B1] Aerts H. J. Velazquez E. R. Leijenaar R. T. Parmar C. Grossmann P. Carvalho S.. (2014). Decoding tumour phenotype by noninvasive imaging using a quantitative radiomics approach. Nat. Commun. 5:4006. 10.1038/ncomms5006 24892406 PMC4059926

[B2] Chang C.-C. Lin C.-J. (2011). LIBSVM: a library for support vector machines. ACM Transact. Intell. Syst. Technol. 2, 1–27. 10.1145/1961189.1961199

[B3] Chen Q. Zhu D. Liu J. Zhang M. Xu H. Xiang Y.. (2021). Clinical-radiomics nomogram for risk estimation of early hematoma expansion after acute intracerebral hemorrhage. Acad. Radiol. 28, 307–317. 10.1016/j.acra.2020.02.021 32238303

[B4] Chen S. Ma K. Zheng Y. (2019). Med3D: Transfer learning for 3D medical image analysis. arXiv [preprint]. 10.48550/arXiv.1904.00625

[B5] Deepak S. Ameer P. (2019). Brain tumor classification using deep cnn features via transfer learning. Comput. Biol. Med. 111:103345. 10.1016/j.compbiomed.2019.103345 31279167

[B6] Deng J. Dong W. Socher R. Li L.-J. Li K. Fei-Fei L. (2009). “ImageNet: a large-scale hierarchical image database,” in 2009 IEEE Conference on Computer Vision and Pattern Recognition (Miami, FL: IEEE), 248–255.

[B7] Fischl B. (2012). Freesurfer. Neuroimage 62, 774–781. 10.1016/j.neuroimage.2012.01.021 22248573 PMC3685476

[B8] Gou S. Tong N. Qi S. Yang S. Chin R. Sheng K. (2020). Self-channel-and-spatial-attention neural network for automated multi-organ segmentation on head and neck ct images. Phys. Med. Biol. 65:245034. 10.1088/1361-6560/ab79c3 32097892

[B9] He K. Zhang X. Ren S. Sun J. (2016). “Deep residual learning for image recognition,” in Proceedings of the IEEE Conference on Computer Vision and Pattern Recognition (Las Vegas, NV), 770–778.

[B10] Jiang L. Zhou L. Yong W. Cui J. Geng W. Chen H.. (2023). A deep learning-based model for prediction of hemorrhagic transformation after stroke. Brain Pathol. 33:e13023. 10.1111/bpa.13023 34608705 PMC10041160

[B11] LeCun Y. Bengio Y. Hinton G. (2015). Deep learning. Nature 521, 436–444. 10.1038/nature14539 26017442

[B12] Lin T.-Y. Dollár P. Girshick R. He K. Hariharan B. Belongie S. (2017). “Feature pyramid networks for object detection,” in Proceedings of the IEEE Conference on Computer Vision and Pattern Recognition (Honolulu, HI), 2117–2125.

[B13] Louis D. N. Perry A. Reifenberger G. Von Deimling A. Figarella-Branger D. Cavenee W. K.. (2016). The 2016 world health organization classification of tumors of the central nervous system: a summary. Acta Neuropathol. 131, 803–820. 10.1007/s00401-016-1545-1 27157931

[B14] Mazya M. Egido J. A. Ford G. A. Lees K. R. Mikulik R. Toni D.. (2012). Predicting the risk of symptomatic intracerebral hemorrhage in ischemic stroke treated with intravenous alteplase: safe implementation of treatments in stroke (sits) symptomatic intracerebral hemorrhage risk score. Stroke 43, 1524–1531. 10.1161/STROKEAHA.111.644815 22442178

[B15] Nielsen A. Hansen M. B. Tietze A. Mouridsen K. (2018). Prediction of tissue outcome and assessment of treatment effect in acute ischemic stroke using deep learning. Stroke 49, 1394–1401. 10.1161/STROKEAHA.117.019740 29720437

[B16] Paciaroni M. Bandini F. Agnelli G. Tsivgoulis G. Yaghi S. Furie K. L.. (2018). Hemorrhagic transformation in patients with acute ischemic stroke and atrial fibrillation: time to initiation of oral anticoagulant therapy and outcomes. J. Am. Heart Assoc. 7:e010133. 10.1161/JAHA.118.010133 30571487 PMC6404429

[B17] Paszke A. Gross S. Massa F. Lerer A. Bradbury J. Chanan G.. (2019). Pytorch: an imperative style, high-performance deep learning library. Adv. Neural Inf. Process. Syst. 32. 10.48550/arXiv.1912.01703

[B18] Seetha J. Raja S. S. (2018). Brain tumor classification using convolutional neural networks. Biomed. Pharmacol. J. 11:1457. 10.13005/bpj/1511

[B19] Sinha A. Dolz J. (2020). Multi-scale self-guided attention for medical image segmentation. IEEE J. Biomed. Health Inf. 25, 121–130. 10.1109/JBHI.2020.2986926 32305947

[B20] Smith S. M. Jenkinson M. Woolrich M. W. Beckmann C. F. Behrens T. E. Johansen-Berg H.. (2004). Advances in functional and structural MR image analysis and implementation as FSL. Neuroimage 23, S208–S219. 10.1016/j.neuroimage.2004.07.051 15501092

[B21] Strbian D. Michel P. Seiffge D. J. Saver J. L. Numminen H. Meretoja A.. (2014). Symptomatic intracranial hemorrhage after stroke thrombolysis: comparison of prediction scores. Stroke 45, 752–758. 10.1161/STROKEAHA.113.003806 24473180

[B22] Tandel G. S. Biswas M. Kakde O. G. Tiwari A. Suri H. S. Turk M.. (2019). A review on a deep learning perspective in brain cancer classification. Cancers 11:111. 10.3390/cancers11010111 30669406 PMC6356431

[B23] Tustison N. J. Avants B. B. Cook P. A. Zheng Y. Egan A. Yushkevich P. A.. (2010). N4ITK: improved N3 bias correction. IEEE Trans. Med. Imaging 29, 1310–1320. 10.1109/TMI.2010.2046908 20378467 PMC3071855

[B24] Vaswani A. Shazeer N. Parmar N. Uszkoreit J. Jones L. Gomez A. N.. (2017). Attention is all you need. Adv. Neural Inf. Process. Syst. 30. 10.48550/arXiv.1706.03762

[B25] Vidal S. M. Chaudhry F. S. Schneck M. (2013). Management of acute ischemic stroke. Hosp. Pract. 41, 108–122. 10.3810/hp.2013.04.1060 23680742

[B26] Virani S. S. Alonso A. Aparicio H. J. Benjamin E. J. Bittencourt M. S. Callaway C. W.. (2021). Heart disease and stroke statistics-2021 update: a report from the american heart association. Circulation 143, e254–e743. 10.1161/CIR.0000000000000950 33501848 PMC13036842

[B27] Wold S. Esbensen K. Geladi P. (1987). Principal component analysis. Chemometr. Intell. Lab. Syst. 2, 37–52. 10.1016/0169-7439(87)80084-9

[B28] Yaniv Z. Lowekamp B. C. Johnson H. J. Beare R. (2018). Simpleitk image-analysis notebooks: a collaborative environment for education and reproducible research. J. Digit. Imaging 31, 290–303. 10.1007/s10278-017-0037-8 29181613 PMC5959828

[B29] Zhang Z. Zhao T. Gay H. Zhang W. Sun B. (2021). Weaving attention U-net: a novel hybrid CNN and attention-based method for organs-at-risk segmentation in head and neck CT images. Med. Phys. 48, 7052–7062. 10.1002/mp.15287 34655077

